# Doppler transcranien au cours de la drépanocytose chez l'enfant Malagasy

**DOI:** 10.11604/pamj.2016.23.264.7072

**Published:** 2016-04-29

**Authors:** Nicolas Fanantenana Herinirina, Lova Hasina Ny Ony Narindra Rajaonarison, Andry Roussel Herijoelison, Olivat Aimée Alson Rakoto, Ahmad Ahmad

**Affiliations:** 1 AService Imagerie Médicale, Centre Hospitalier Universitaire d'Antsiranana, Madagascar; 2 Service Imagerie Médicale, Centre Hospitalier Universitaire Joseph Ravoahangy Andrianavalona, Antananarivo, Madagascar; 3 Service Imagerie Médicale, Centre Hospitalier Universitaire de Toamasina, Madagascar; 4 Service Hématologie et Biologie, Centre Hospitalier Universitaire Joseph Ravoahangy Andrianavalona, Antananarivo, Madagascar

**Keywords:** Accident vasculaire cérébrale, doppler transcrânien, drépanocytose, enfant, Cerebrovascular accident, transcranial Doppler, sickle cell disease, child

## Abstract

**Introduction:**

Le doppler transcrânien est un outil efficace permettant de dépister les enfants drépanocytaires à risque d'AVC.

**Méthodes:**

Nous avons réalisé une étude descriptive transversale sur des enfants Malagasy âgés entre 24 mois et 15 ans (groupe 1: 57 drépanocytaires, groupe 2: 43 témoins) afin d’évaluer le profil vélocimétrique des artères cérébrales chez les drépanocytaires. Un examen Doppler transcrânien a été réalisé avec étude des flux sanguins cérébraux chez les enfants des deux groupes.

**Résultats:**

Pour les sujets drépanocytaires, la vitesse moyenne (VM) de l'artère cérébrale moyenne était de 100,9 ± 26,8 cm/s, l'indice de pulsatilité (IP) de 0,73 ± 0,20, la différence entre les artères cérébrales moyennes droite et gauche (ACMr) de 19,8 ± 21,5 cm/s, le rapport des vitesses de l'artère cérébrale antérieure/artère cérébrale moyenne (ACA/ACM) de 0,7 ± 0,2. Pour les enfants non drépanocytaires, VM: 80,6 ± 19,3 cm/s, IP: 0,79 ± 0,14, ACMr: 17 ± 20,1 cm/s, ACA/ACM: 0,8 ± 0,2. La vélocité des enfants drépanocytaires était supérieure au groupe contrôle. Les vitesses ont été corrélées avec le taux d'hémoglobine et l’âge et non pas avec le sexe et le volume globulaire moyen.

**Conclusion:**

Les vitesses circulatoires cérébrales sont élevées chez les drépanocytaires que les enfants non drépanocytaires et sont influencées par le taux d'hémoglobine et l’âge.

## Abstract (en)

**Introduction:**

Transcranial Doppler is an effective diagnostic tool to detect sickle cell children at risk for stroke.

**Methods:**

We conducted a cross-sectional descriptive study of Malagasy children aged between 24 months and 15 years (group 1: 57 sickle cell diseases, group 2: 43 controls) to assess the cerebral artery velocimetry in the patient population. Transcranial doppler ultrasound with analysis of cerebral blood flow was performed in children of both groups.

**Results:**

In subjects with sickle cell disease, the mean velocity (MV) in the middle cerebral artery was 100.9 ± 26.8 cm/s, pulsatility index (PI) was 0.73 ± 0.20, the difference between the right and left middle cerebral artery (MCA) was 19.8 ± 21.5 cm/s, anterior cerebral artery/middle cerebral artery flow velocity ratio (ACA / MCA) was 0.7 ± 0.2. In children without sickle cell disease, MV: 80.6 ± 19.3 cm/s, IP: 0.79 ± 0.14, ACMr: 17 ± 20.1 cm/s, ACA/ACM: 0,8 ± 0,2. The velocity in children with sickle cell disease was higher than in the control group. Velocities were correlated with hemoglobin and age but not with sex and mean corpuscular volume.

**Conclusion:**

Cerebral Circulation Speeds are higher in children with sickle cell disease and are influenced by hemoglobin levels and age.

## Introduction

La drépanocytose est une vasculopathie sténosante progressive avec comme principales conséquences des lésions d'ischémie et d'infarctus tissulaire et un risque élevé d'accident vasculaire cérébral ischémique [1]. Une détection précoce de ces vasculopathies à l'aide d'une échographie Doppler transcranienne est indispensable pour éviter des séquelles neurologiques graves ou même une issue fatale. Notre étude avait pour objectif d’établir le profil vélocimétrique des artères intracérébrales des enfants drépanocytaires Malagasy.

## Méthodes

Il s'agit d'une étude descriptive et analytique, transversale entre deux populations d'enfants drépanocytaires et non drépanocytaires âgés entre 24 mois et 15 ans qui s'est déroulée au sein du service d'Imagerie Médicale du Centre Hospitalier Universitaire Ravoahangy Andrianavalona, Antananarivo, Madagascar allant du 08 février 2012 au 23 juillet 2012, soit une période de 6 mois. L'autorisation orale des parents des enfants et ou l'assentiment des enfants participants ont été obtenu après explication des objectifs et du déroulement de l’étude. Nous avons inclus dans le groupe 1, le quart des enfants drépanocytaires de phénotype homozygote SS recensés et confirmés biologiquement, ne recevant pas de traitement transfusionnel sanguin durant la période d’étude, en utilisant un échantillonnage aléatoire stratifié (garçon et fille). Dans le groupe 2, des enfants non drépanocytaires, en bonne santé apparente, comprenant des garçons et des filles ont été recrutés selon un échantillonnage aléatoire stratifié. Nous avons exclu les enfants drépanocytaires de phénotype hétérozygote, les enfants non drépanocytaires présentant une tare particulière. L’échographe utilisé était un appareil de marque Mindray DC6 équipé d'une sonde sectorielle de 2 MHz. Nous avons étudié chez tous les enfants la vitesse moyenne des flux des artères cérébrales, l'index de pulsatilité, la différence entre la vitesse de l'artère cérébrale moyenne droite et gauche, le rapport entre les vitesses de l'artère cérébrale antérieure et de l'artère cérébrale moyenne, la corrélation des vélocités avec le taux d'hémoglobine, le volume globulaire moyen, l’âge et le sexe. Les données ont été anonymisées selon le principe du Comité National d'Ethique. La saisie des données a été faite sur logiciel Microsoft Excel 2007 de Windows suivie de l'analyse statistique des données sur le logiciel Epi-Info 3.5.3. Le test d'indépendance du Khi-carré de Pearson et le test t de Student ont été utilisés pour analyser et comparer les deux groupes. Le degré de signification utilisé pour déterminer une différence statistiquement significative est p < 0,05.

## Résultats

**Figure 1:**
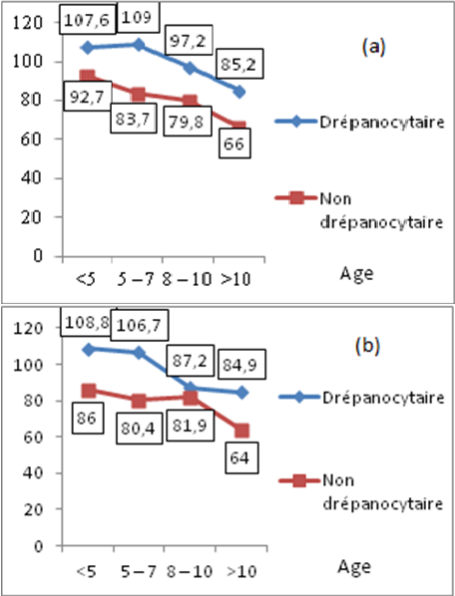
Evolution de la vitesse mesurée à droite (a) et à gauche (b) en fonction de l’âge chez les deux groupes de population

Cette étude regroupait 100 enfants âgés de 24 mois à 15 ans avec un âge moyen de 7,40 ans constituant 57 filles et 43 garçons. Le groupe 1 est constitué de 57 enfants drépanocytaires dont 25 garçons et 32 filles avec un âge moyen de 7,08 ans. Le groupe 2 est constitué de 43 enfants non drépanocytaires dont 18 garçons et 25 filles avec un âge moyen de 8,53 ans. Plus de la moitié (52,6%) des enfants drépanocytaires avaient un taux d'hémoglobine (Hb) entre 61 et 80 g/dl, 3 enfants ont présenté une anémie sévère (taux d'Hb inférieur à 60 g/dl), pour le reste 22 sujets avaient un taux d'Hb entre 80 et 100 g/dl et 2 autres un taux d'Hb supérieur à 100 g/dl. Dans cette série, 35 (61,4%) drépanocytaires avaient un taux de VGM entre 81 et 100 µ^3^, 13 sujets avec taux de VGM inférieur à 80 µ^3^ et 9 sujets avec un taux de VGM supérieur à 100 µ^3^. La vitesse moyenne mesurée au niveau des différentes artères intracérébrales droite et gauche est plus accélérée chez les drépanocytaires par rapport aux non drépanocytaires (Tableau 1). Les enfants drépanocytaires présentent un indice de pulsatilité globalement plus bas que les non drépanocytaires (Tableau 2). La différence chiffrée en cm/s des valeurs des vitesses moyennes des ACM droite et gauche se situe approximativement aux alentours de 20 (Tableau 3). Le rapport des vitesses moyennes de l'artère cérébrale antérieure sur l'artère cérébrale moyenne est de 0,7 ± 0,2 chez les drépanocytaires, il est de 0,8 ± 0,2 chez les non drépanocytaires (Tableau 4). Les vitesses moyennes varient significativement avec le taux d'hémoglobine, les vitesses les plus élevées sont constatées avec les taux d'hémoglobine les plus bas. La vitesse moyenne est donc inversement proportionnelle au taux d'hémoglobine. La valeur statistique de p est de 0,01 à droite et 0,02 à gauche (Tableau 5). La vitesse mesurée à droite et à gauche est inversement proportionnelle à l’âge chez les deux groupes de population (Figure 1). La vitesse moyenne de l'artère cérébrale moyenne ne varie pas suivant le sexe, p-value est non significative chez les deux groupes de populations (Tableau 6). On trouve l'absence de variation de la vitesse moyenne de l'ACM suivant le taux du volume globulaire moyen. La valeur statistique de « p » est non significative (Tableau 7).

**Tableau 1:** Valeur comparative de la vitesse moyenne entre les deux groupes de population

	VITESSE MOYENNE (cm/s) [Moyenne ± Ecart Type (min – max) n: effectif]			
	A DROITE		A GAUCHE	
	Drépanocytaire	Non drépanocytaire	Drépanocytaire	Non drépanocytaire
**ACM**	**100,9 ± 26,8** (49,8 – 159,6) 44	**80,6 ± 19,3** (42,7 – 113,8) 35	**98 ± 25,6** (40,5 – 158,4) 49	**78,5 ± 19** (31,9 – 119,8) 38
**ACA**	**76,9 ± 20,7** (27,3 – 138,8) 35	**66,7 ± 20,5** (27,7 – 105,4) 17	**71,5 ± 22,8** (43,4 – 142) 28	**65,4 ± 17,3** (24 – 100,1) 17
**ACI**	**76,2 ± 29,3** (19,9 – 150,2) 43	**49,4 ± 14,8** (28,1 – 83,4) 31	**65,7 ± 24,3** (18 – 129) 41	**51,1 ± 18,3** (25,5 – 99,6) 31
**ACP**	**51,9 ± 20,6** (21 – 154,3) 52	**33,9 ± 9,9** (17,9 – 58,5) 35	**51,1 ± 23,8** (12,5 – 133,8) 52	**34,2 ± 10,7** (14,3 – 55,5) 34
**TB**	**65,9 ± 19,6** (30,6 – 115) 48	**44,9 ± 11,6** (26–70) 25		

Note: ACM: artère cérébrale moyenne; ACA: artère cérébrale antérieure; ACI: artère carotide interne; ACP: artère cérébrale postérieure; TB: tronc basilaire

**Tableau 2:** Valeurs de l'indice de pulsatilité chez les deux groupes d'enfants

	INDICE DE PULSATILITE [Moyenne ± Ecart Type (min – max) n: Effectif]			
	A DROITE		A GAUCHE	
	Drépanocytaire	Non drépanocytaire	Drépanocytaire	Non drépanocytaire
**ACM**	**0,73 ± 0,20** (0,48 – 1,31) 44	**0,79 ± 0,14** (0,48 – 1,21) 35	**0,73 ± 0,14** (0,46 – 1,07) 49	**0,83 ± 0,15** (0,58 – 1,22) 38
**ACA**	**0,69 ± 0,14** (0,35 – 1,05) 35	**0,77 ± 0,17** (0,52 – 1,03) 17	**0,68 ± 0,13** (0,42 – 1,00) 28	**0,79 ± 0,09** (0,62 – 0,97) 17
**ACI**	**0,74 ± 0,14** (0,46 – 1,07) 43	**0,84 ± 0,11** (0,68 – 1,14) 31	**0,72 ± 0,15** (0,39 – 1,14) 41	**0,86 ± 0,15** (0,58 – 1,27) 31
**ACP**	**0,68 ± 0,11** (0,44 – 0,94) 52	**0,80 ± 0,14** (0,53 – 1,12) 35	**0,68 ± 0,20** (0,22 – 1,37) 52	**0,80 ± 0,13** (0,56 – 1,09) 34
**TB**	**0,56 ± 0,14** (0,28 – 0,95) 48	**0,73 ± 0,21** (0,41 – 1,23) 25		

Note: ACM: artère cérébrale moyenne; ACA: artère cérébrale antérieure; ACI: artère carotide interne; ACP: artère cérébrale postérieure; TB: tronc basilaire

**Tableau 3:** Différence entre les vitesses des artères intracrâniennes à droite et à gauche des deux groupes de population

	DIFFERENCE (cm/s) [Moyenne ± ET (min – max) n: Effectif]		P-value
	DREPANOCYTAIRE	NON DREPANOCYTAIRE	
**ACM**	**19,8 ± 21,5** (0,4 – 44,8) 44	**17 ± 20,1** (0,5 – 42,8) 35	0,29
**ACA**	**29,4 ± 34** (5,7 – 65,3) 23	**23,4 ± 24,5** (0,2 – 43,3) 12	0,38
**ACI**	**27,4 ± 39,7** (2 – 95,1) 34	**24 ± 29,8** (1,1 – 63,4) 27	0,61

Note: ACM: artère cérébrale moyenne; ACA: artère cérébrale antérieure; ACI: artère carotide interne

**Tableau 4:** Rapport des vitesses moyennes ACA/ACM des deux groupes de population

	Vitesse ACA/ACM [Moyenne ± ET (min – max) n: Effectif]		
	DREPANOCYTAIRE	NON DREPANOCYTAIRE	P-value
**A DROITE**	**0,7 ± 0,2** (0,2 – 1,2) 32	**0,8 ± 0,2** (0,3 – 1,3) 17	**0,29**
**A GAUCHE**	**0,7 ± 0,2** (0,4 – 1,2) 27	**0,8 ± 0,2** (0,3 – 1,1) 17	**0,15**

**Tableau 5:** Relation entre le taux d'hémoglobine et la vitesse moyenne de l'ACM des patients drépanocytaires

TAUX HEMOGLOBINE (g/dl)	VITESSE MOYENNE (cm/s) [Moyenne ± ET (min – max) n: Effectif]	
	DROITE (P=0,01)	GAUCHE (p=0,02)
**<60**	**140,8 ± 3,53** (138,3 – 143,3) 2	**130,2 ± 34,4** (105,9 -245,6) 2
**61 – 80**	**108,1 ± 29,8** (49,8 – 159,6) 22	**104,7 ± 23,2** (70,1 – 158,4) 26
**81 – 100**	**88,7 ± 16,9** (65,6 – 136) 18	**86 ± 24,6** (40,5 – 146,2) 19
**>100**	**90,7 ± 10,4** (83,3 – 98,1) 2	**92,4 ± 5,5** (88,5 – 96,4) 2

**Tableau 6:** Variation de la vitesse moyenne de l'ACM par rapport au sexe, chez les deux populations à droite et à gauche

	VITESSE MOYENNE A DROITE (cm/s) [Moyenne ± ET (min – max) n: Effectif]	
GENRE	DREPANOCYTAIRE (P=0,97)	NON DREPANOCYTAIRE (p=0,37)
**MASCULIN**	101,1 ± 20,7 (72,9 – 147,4) 16	76,8 ± 14,7 (48,9 – 98,5) 13
**FEMININ**	100,8 ± 30 (49,8 – 159,6) 28	82,9 ± 21,5 (42,7 – 113,8) 22
	**VITESSE MOYENNE A GAUCHE (cm/s)** [Moyenne ± ET (min – max) n: Effectif]	
**GENRE**	**DREPANOCYTAIRE (p=0,99)**	**NON DREPANOCYTAIRE (p=0,18)**
**MASCULIN**	**98 ± 27** (60,5 – 158,4) 21	**73,4 ± 18,3** (31,9 – 105,5) 15
**FEMININ**	**97,9 ± 25** (40,5 – 154,6) 28	**81,9 ± 19** (50,9 – 119,8) 23

**Tableau 7:** Variation de la vitesse moyenne de l'ACM selon le volume globulaire moyen

	VITESSE MOYENNE (cm/s) [Moyenne ± ET (min – max) n: Effectif]	
VGM (µ^3^)	A DROITE (p=0,50)	A GAUCHE (p=0,98)
**<80**	**109,7 ± 29,9** (76,1 – 159,6) 10	**98,2 ± 27,6** (68,7 – 158,4) 11
**81 – 100**	**98,1 ± 26** (49,8 – 150) 26	**97,5 ± 25,9** (40,5 – 154,6) 29
**>100**	**98,9 ± 26,1** (61,1 – 136) 8	**99,2 ± 25,3** (68 – 146,2) 9

## Discussion

Malgré l'effectif restreint de la population étudiée, un profil vélocimétrique concernant la vitesse moyenne des grosses artères intracérébrales des enfants drépanocytaires et non drépanocytaires malagasy a pu être dégagé. La vitesse moyenne et la différence des vitesses moyennes à droite et à gauche des enfants drépanocytaires sont nettement supérieures à celles des enfants non drépanocytaires dans notre étude comme dans la littérature [1, 2]. L'indice de pulsatilité est discrètement inférieur chez nos patients drépanocytaires. Même si la différence est minime, cette constatation s'observe sur la totalité des troncs artériels étudiés. Melo et al ont trouvé également une différence significative des indices de pulsatilité entre les deux groupes de population étudiée [1]. Ce qui est aussi en rapport avec les valeurs de références fournies par Babikian [3]. Concernant le rapport des vitesses ACA/ACM, nos chiffres sont pratiquement identiques à ceux de Melo, il est de 0,7 ± 02 pour les enfants drépanocytaires et de 0,8 ± 02 pour les enfants non drépanocytaires [1]. Sa valeur normale est de 1,2; un rapport supérieur à 1,2 correspond soit à une accélération majeure de la vitesse de l'artère cérébrale antérieure soit à un ralentissement anormal de la vélocité de l'artère cérébrale moyenne faisant craindre un état pré-occlusif avec risque d'ischémie ou d'accident vasculaire [3]. On retrouve les mêmes observations que celles qui ont été publiées dans la littérature concernant cette différence acceptable entre les valeurs des vitesses moyennes enregistrées au niveau des deux côtés droit et gauche des artères intracérébrales. Melo et al ont trouvé un ratio de 14,53 ± 15,23 au niveau de l'artère cérébrale moyenne des enfants drépanocytaires et un ratio de 13,19 ± 13,77 chez les enfants non drépanocytaires [1]. Au delà de 50% de différence ou d'un ratio droite/gauche ou gauche/droite inférieure à 0,5, le risque d'AVC est très grande [3]. Il existe une corrélation statistiquement significative entre la valeur de la vitesse moyenne et le taux d'hémoglobine. Ce qui vient confirmer les données déjà évoquées par la littérature c'est à dire, la vitesse moyenne est inversement proportionnelle au taux de l'hémoglobine [4–7]. Les résultats observés sur l’évolution de la vitesse moyenne et de la vitesse systolique maximale au niveau de l'artère cérébrale moyenne par rapport à l’âge sont relativement superposable avec les données des autres études [1, 3, 8]. Notre étude se confond avec les autres publications concernant l'absence de corrélation entre la valeur de la vitesse moyenne et le sexe. Le volume globulaire moyen ne varie pas avec les valeurs de la vitesse moyenne au niveau de l'artère cérébrale moyenne, ce qui est compatible avec les autres études [1, 3, 8]. Néanmoins, Verlhac propose qu'un volume globulaire inférieur à 75 µ3 confirme l'absence de risque d'une vitesse supérieure à 200 cm/s [8].

Dans notre étude, les données comparatives entre les deux groupes de population d'enfant drépanocytaires et non drépanocytaires convergent exactement avec celles des autres études. Chez les enfants drépanocytaires malagasy, on note une valeur de vitesses nettement supérieure à celles des enfants non drépanocytaires. Ces données constituent une valeur de référence à laquelle on peut se rapporter pour se repérer quant aux valeurs de vitesse normale des enfants malagasy. Par contre, il existe une corrélation entre la vélocité et le volume globulaire moyen selon la littérature [5]. Ceci s'explique probablement par la normalité de tous nos résultats chez les deux groupes de population et le bon état de santé apparente de nos sujets.

## Conclusion

La vélocité du flux des artères cérébrales des enfants drépanocytaires est supérieure à celle des enfants non drépanocytaires. Il existe une corrélation entre la vitesse et le taux d'hémoglobine et l’âge; et non pas avec le sexe et le volume globulaire moyen. Le programme de dépistage utilisant le Doppler transcrânien peut être appliqué au niveau de la population pédiatrique Malagasy.

### Etat des connaissances actuelle sur le sujet

- Vasculopathie sténosante progressive;
- Augmentation des vitesses en cas de sténose;
- Risque d'AVC augmenté en fonction des vitesses.

### Contribution de notre étude à la connaissance

- Vitesses circulatoires cérébrales des enfants drépanocytaires supérieures à celle des enfants non drépanocytaires;
- Le taux de l'hémoglobine et l’âge influencent sur les vitesses;
- Le genre et le volume globulaire n'influence pas la vitesse circulatoire.
